# Factors Influencing the Mortality of Patients with Subarachnoid Haemorrhage in the Intensive Care Unit: A Retrospective Cohort Study

**DOI:** 10.3390/jcm14051650

**Published:** 2025-02-28

**Authors:** Onur Cetinkaya, Ulku Arslan, Hakan Temel, Ali Sait Kavakli, Hakan Cakin, Melike Cengiz, Murat Yilmaz, Nur Ebru Barcin, Fatih Ikiz

**Affiliations:** 1 Department of Anesthesiology and Reanimation, Akdeniz University Faculty of Medicine, Antalya 07070, Turkey; onurcetinkaya94@gmail.com (O.C.); hakantemel29@yahoo.com.tr (H.T.); melikecengiz@yahoo.com (M.C.); muryigit@yahoo.com (M.Y.); 2 Department of Anesthesiology and Reanimation, Istinye University Faculty of Medicine, Istanbul 34010, Turkey; alisaitkavakli@hotmail.com; 3 Department of Neurosurgery, Akdeniz University Faculty of Medicine, Antalya 07070, Turkey; hcakin@akdeniz.edu.tr; 4 Department of Neurology, Akdeniz University Faculty of Medicine, Antalya 07070, Turkey; ebrubarcin@gmail.com; 5 Department of Emergency Medicine, Konya Beyhekim Training and Research Hospital, Konya 42130, Turkey; sultanmehmet01@hotmail.com

**Keywords:** intensive care unit, mortality, spontaneous subarachnoid haemorrhage

## Abstract

**Background:** Spontaneous subarachnoid haemorrhage (SAH) represents a significant cerebrovascular disease with considerable morbidity and mortality. The aim of this study was to determine the demographic/clinical characteristics of spontaneous SAH patients admitted in the intensive care unit (ICU) and factors affecting the mortality. **Methods:** This study was designed as a retrospective cohort study that included patients with a diagnosis of spontaneous SAH hospitalized in the ICU. The clinical and radiological parameters were compared between mortality and survival cohorts. Univariate logistic regression analyses were performed for the effect profiles of the parameters on mortality. **Results:** ICU mortality was 41% in patients with spontaneous SAH. A number of factors have been identified as being independently associated with mortality in the studied cohort. These factors are hospital admission with loss of consciousness (Glasgow Coma Scale score <8), a high Clinical Comorbidity Index score, stage >2 according to the Hunt and Hess grading system and complication status (meningitis and sepsis/septic shock). **Conclusions:** Spontaneous SAH is a condition associated with a high mortality in severe cases. Patients exhibiting these risk factors require meticulous monitoring in the ICU.

## 1. Background

Spontaneous subarachnoid haemorrhage (SAH) represents a significant cerebrovascular disease with considerable morbidity and mortality [1]. It is typically managed in specialized centres with neurological intensive care unit (ICU) facilities. Spontaneous SAH is a well-known disease with an incidence of 7.2–9.0 per 100,000 per year, largely due to aneurysm rupture [2]. It has been reported that the mortality of aneurysmal SAH is as high as 50% [3]. Beyond high morbidity and mortality, it is also associated with a prolonged hospital stay [4]. Furthermore, it places an additional economic burden on health systems, affecting the young population (50 to 60 years old) and resulting in a longer length of stay in hospital and ICU [5].

Despite the evidence from numerous clinical studies indicating that early intervention and multidisciplinary ICU management can enhance the clinical outcomes of patients with spontaneous SAH, mortality and morbidity remain significant concerns [6,7,8]. Moreover, algorithms for ICU management in this patient group have been developed and consensus guidelines have been published [9,10]. However, there are still uncertainties regarding the improvement of mortality in critically ill patients requiring ICU. Determining the factors associated with mortality in the ICU follow-up is very important for clarifying these uncertainties and improving ICU outcomes. The number of studies analysing ICU mortality and mortality-related factors in this patient group is limited. Furthermore, it has been shown that there are regional differences in the incidence of SAH [11]. Mortality and factors influencing mortality are also likely to differ between regions and centres. A large study in the United States examining the prognostic effect of gender, race and ethnicity in patients with aneurysmal SAH reported that white patients were more likely to have worse clinical outcomes [12]. Data on the characteristics and prognosis of Turkish patients with spontaneous SAH that are admitted to ICU are poor. The aim of this study was to determine the demographic/clinical characteristics of spontaneous SAH patients admitted in the ICU and factors affecting the mortality.

## 2. Methods

### 2.1. Study Design

A retrospective cohort study was conducted in the 33-bed general ICUs of Akdeniz University Faculty of Medicine Hospital, Antalya, Turkey. The study was carried out in accordance with the Declaration of Helsinki and approved by the Ethics Committee of Akdeniz University Faculty of Medicine (approval no: TBAEK-89, dated 5 March 2024). In addition, this study has been retrospectively registered in the Clinicaltrials.gov clinical trials registry (no. NCT06490640, dated 29 June 2024). The sample included patients with spontaneous SAH who were admitted in the ICU between 01 January 2019 and 31 December 2023. Data of the patients were obtained from patient observation files and hospital information system database. Due to the retrospective nature of the study, the requirement for informed consent was waived. The investigators analysed only anonymised data.

### 2.2. Participants

Patients aged 18 years and older who were diagnosed with spontaneous SAH and admitted to the ICU were included in the study. The diagnosis of spontaneous SAH was based on brain-computed tomography (CT) or lumbar puncture. Patients with a final outcome (survival or mortality) in our centre were included in the analysis. Only patients admitted to the ICU for the first time were included. Patients with traumatic SAH, history of significant head trauma in the previous two weeks (any abnormality on brain CT requiring hospitalization for more than 24 h), pregnancy and length of ICU stay ≤24 h were excluded.

### 2.3. Management of Intensive Care Unit

Because of the need for close neurological and haemodynamic monitoring in the early period (first 72 h) in patients with spontaneous SAH, all patients presenting to the emergency department were admitted to the ICU. In the postoperative period, patients who could not be extubated, had uncontrollable seizures, had respiratory or haemodynamic instability, required mechanical ventilation, or had a Glasgow Coma Scale (GCS) score of ≤8 were also admitted to ICU. The clinical and radiological severity of patients at ICU admission was determined according to Acute Physiological and Chronic Health Evaluation II score (APACHE II), GCS, Modified Fisher Scale, World Federation of Neurological Societies (WFNS) and Hunt and Hess (H&H) grading systems. Patients with severe agitation despite mild sedation and pain control, a GCS score ≤8 and inadequate airway protection were intubated, mechanically ventilated and sedated [10]. All patients underwent CT angiography and/or digital subtraction angiography for the aetiology within the first 24 h. The decision regarding the aetiological treatment was made by faculty members from the departments of interventional radiology and neurosurgery, in accordance with the treatment guidelines that were current at the time. Endovascular coiling was preferred in posterior circulation aneurysms. Furthermore, the aetiological treatments were performed by the same neurosurgeon and interventional radiologist. The treatments were performed within the first 72 h. Blood pressure was controlled to avoid hypotension, and vasopressors (norefineprin) were administered in patients who did not achieve the target mean arterial blood pressure of 65 mmHg. Patients received fluid therapy to maintain euvolaemia. Intracranial pressure was evaluated daily by bedside ultrasonography and optic nerve sheath diameter measurement by an ICU specialist with at least two years of experience. Measurements were repeated in case of clinical necessity or doubt. An optic nerve sheath diameter of ≥5 mm was associated with increased intracranial pressure [13]. Brain CT imaging was performed to determine conditions such as acute hydrocephalus, rebleeding, diffuse cerebral oedema and cerebral ischaemia that may cause increased intracranial pressure. In patients with clinical and radiological findings compatible with increased intracranial pressure, an extraventricular/lumbar drainage (EVD/LD) catheter was placed with or without decompressive craniotomy. In addition, osmotic agents (20% mannitol, 3% NaCl) and loop diuretics (furasemide) were administered to these patients. Continuous intracranial pressure monitoring was performed in patients with EVD. Sudden clinical deterioration (acute neurological regression, seizure, bradycardia or sudden hypertensive blood pressure) with expansion of bleeding on brain CT or sudden clinical deterioration with fresh blood in the EVD/LD catheter were defined as rebleeding [14]. Daily transcranial Doppler ultrasonography monitoring was performed. Vasospasm was defined as a middle cerebral artery mean flow velocity divided by the ipsilateral extracranial internal carotid artery ratio above 3 and/or a middle cerebral artery velocity above 120 cm/sec in the presence of new focal or global neurological disorders that could not be explained by other pathologies [15]. An amount of 60 mg of Nimodipine was administered every four hours per standard therapy. Patients with vasospasm in whom the risk of rebleeding was not high were given vasopressor support (norepinephrine) and controlled hypertension was applied with a target systolic blood pressure level of 160–180 mmHg. Balloon angioplasty or intra-arterial vasodilators were not used to treat vasospasm in any case. The occurrence of new cerebral infarcts on brain CT in patients with motor limb weakness, aphasia or a decrease of ≥2 points in GCS score during ICU follow-up was considered as delayed cerebral ischaemia (DCI) [16]. Microbiological culture sampling was performed on samples obtained from patients with fever. Meningitis is suspected in patients with a fever higher than 38 °C, headache, nuchal rigidity, meningeal signs and cranial nerve involvement symptoms who did not have any other suspicious infection source. Meningitis is defined as cerebrospinal fluid culture positivity and/or the presence of at least one of the following conditions: increased white blood cell count, increased protein and/or decreased glucose in the cerebrospinal fluid sample, the presence of microorganisms in gram staining, and positive blood culture [17]. Ventilator-associated pneumonia (VAP) is defined as the presence of respiratory symptoms compatible with pneumonia and new radiological changes in a patient who has been mechanically ventilated for 48 h or longer [18]. Sepsis-3 criteria were used for the diagnosis of sepsis/septic shock [19]. All patients were monitored for electrolyte imbalance and serum sodium levels <135 mmol/L were defined as hyponatremia and >145 mmol/L as hypernatremia. Deep vein thrombosis prophylaxis with pneumatic compression was performed in all patients. Low molecular weight heparin was added when possible. Antiepileptic drug treatment was not routinely given to all patients, but was administered to patients who developed seizures. All patients received pain control and stress ulcer prophylaxis.

### 2.4. Data Collection

Demographic and clinical data that had been derived and analysed included age, sex, smoking history, and comorbidities. Presenting signs and symptoms, diagnoses responsible for spontaneous SAH (aneurysm, arterial malformation, etc.), site of aneurysm, risk factors, location of the aneurysm, systolic blood pressure during ICU admission, and scores from grading systems indicating clinical and radiological severity of the disease were recorded. Procedures for spontaneous SAH, complications during ICU follow-up (vasospasm, meningitis, electrolyte disturbances, rebleeding, DCI, VAP, sepsis/septic shock) and treatments, duration of ICU stay and mechanical ventilation, ICU outcome (survival or mortality), causes of mortality and brain death status were recorded. According to the outcome of the ICU process, the patients were divided into two groups—survival and mortality.

### 2.5. Outcome

The primary outcome of the study is to determine mortality and its causes in patients with spontaneous SAH in the ICU. The secondary outcome is to evaluate the clinical characteristics and factors affecting mortality in spontaneous SAH patients.

### 2.6. Statistical Analysis

The data collected in the study were recorded by the principal investigator and subsequently transferred to the statistical software package SPSS version 24 (SPSS Inc., Chicago, IL, USA) for analysis. The data were expressed in both categorical and quantitative forms. Continuous variables were expressed as mean ± standard deviation or median values with minimum, maximum, and interquartile range (IQR). Categorical variables were expressed as number and percentage. The suitability of the data for normal distribution was evaluated using the Kolmogorov–Smirnov test. In instances where normal distribution was achieved, independent and dependent quantitative data were analysed using Student’s *t*-test, while independent qualitative data were analysed using the Pearson chi-square test. Mann–Whitney U test for independent quantitative data and Wilcoxon test for dependent quantitative data were used when normal distribution was not provided. Chi-square or Fisher’s exact tests were used for relationships between categorical parameters for outcome comparisons between survivors and non-survivors. Yates correction was used for appropriate crossover. A receiver operating characteristic (ROC) analysis was conducted to ascertain the discriminative efficacy of the parameters in predicting mortality. The findings of this analysis are presented with the area under the curve (AUC), cut-off points, sensitivity and specificity values, and 95% confidence intervals. The optimal cut-off points for the parameters were calculated using the Youden index. Univariate logistic regression analysis was performed to determine the independent risk factors associated with mortality, and the results presented with odds ratio (OR) and 95% confidence intervals. Due to the fact that the sample size was insufficient and the assumptions of the related analysis were not met, multivariate logistic regression was not performed with the original database. As an alternative method, and in order to carry out a multivariate logistic regression analysis, a virtual database (with a sample size of 1000) has been generated with a non-parametric bootstrapping method (creation has been carried out taking into account the original database structure). The virtual database was generated in the Google Colab platform using the Python (Version 3.13.2) programming language with the implementation of the IIDBootstrap, pandas, and numpy libraries. In our study, the alpha level was set at 0.05 (5%), and *p*-values less than 0.05 were considered statistically significant.

## 3. Results

**Figure 1:**
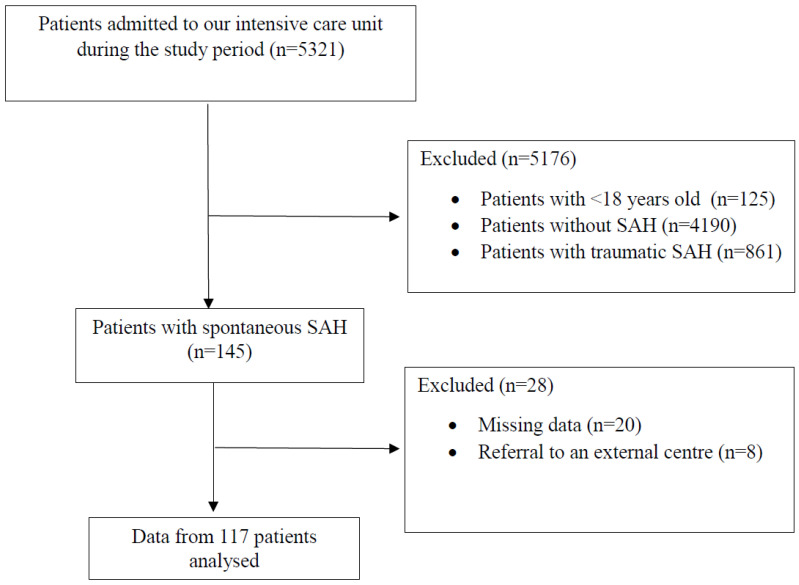
Flow diagram of the study. SAH: subarachnoid haemorrhage.

During the study period, 121 patients with spontaneous SAH were admitted to the ICU and data from 117 patients who met the inclusion criteria were analysed (Figure 1).

Among the study patients, 69 (59%) survived (survival cohort), and 48 (41%) died (mortality cohort). Clinical and demographic characteristics of patients are presented in Table 1. The mean age of the patients was 54.9 ± 15.1 years and 55.6% were male. The majority of the patient population (58.1%) was aged 50 years and older. The mean age of female and male patients was 50.8 ± 5.4 and 57.8 ± 4.3 years, respectively. The median value of the Charlson Comorbidity Index (CCI) score was 1 (0–9) and hypertension was present as comorbidity in 64.1% of the patients. The median value of mean systolic blood pressure at ICU admission was 140 (130–165) mmHg and 57.2% of the patients had increased systolic blood pressure (≥140 mmHg). The most common admission symptoms were headache (76.1%), loss of consciousness (32.5%) and nausea and vomiting (23.9%). In the mortality cohort, the symptom of presentation with headache was lower and loss of consciousness was significantly higher (*p* < 0.001, *p* < 0.001). The median time from admission to the health centre to admission to the ICU was 7 (4–11) hours and was similar between cohorts (*p* = 0.108). The patients were monitored in the intermediate care unit, which was situated within the emergency department, until such time as they were transferred to the ICU.

Radiological data of the patients are presented in Table 2. Angiographic examination for the aetiology was positive in 72 (61.5%) patients (the aetiological factor was determined) and the rate of positive angiographic examination was significantly higher in the group of patients with mortality (*p* = 0.032). A proportion of 48.4% of patients were at Modified Fisher Scale stage 4 and this group of patients was significantly higher in the mortality cohort (*p* < 0.001). The scores obtained from the rating systems are presented in Table 3. In the mortality cohort, the GCS score was lower and the APACHE II, WFNS, H&H scores were higher (*p* < 0.001, *p* < 0.001, *p* < 0.001, *p* < 0.001, *p* < 0.001).

**Figure 2:**
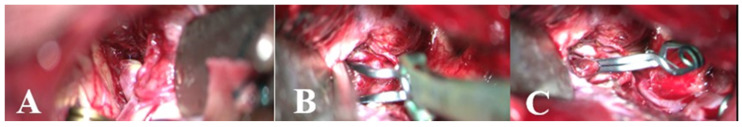
Aneurysm of the anterior cerebral artery (**A**), surgical microclip procedure (**B**), aneurysm following microclip procedure (**C**).

**Figure 3:**
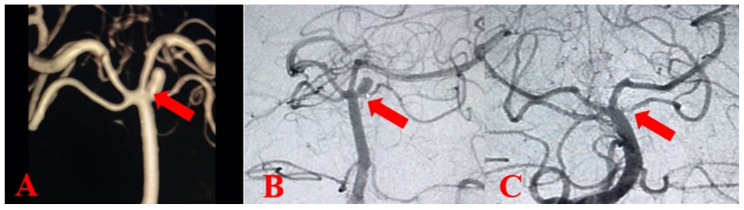
Intracranial aneurysm brain-computed tomography image (**A**), digital subtraction angiography image of intracranial aneurysm (**B**), intracranial aneurysm after endovascular coil procedure (**C**).

Neurosurgery was required in 79 patients. Of the 65 patients with aneurysms, 59 were treated with surgical microvascular clips and 6 with endovascular coil intervention (Figure 2 and Figure 3). Endovascular coil intervention was performed in four patients with aneurysms in the posterior system and in two patients whose general condition was deemed unsuitable for surgical intervention. Patient distribution was similar between the cohorts. The complications detected are presented in Table 4. Vasospasm developed in 45 (38.5%) patients and controlled hypertension could be applied in only 12 (10.3%) patients because of the high risk of rebleeding. In 19 cases, rebleeding was detected, with coagulation disorder identified as the underlying cause in 10 patients and uncontrolled high systolic blood pressure cited as the cause in 6 cases. In three cases, no underlying cause was identified. During ICU follow-up, 82 (70%) patients required mechanical ventilation and the mean duration of mechanical ventilation was 14.1 ± 22 days. Tracheostomy was performed in 30 (25.6%) patients who needed mechanical ventilation. Microbiological examination revealed culture positivity in 59 patients. The most prevalent microorganisms identified were Acinetobacter baumannii (30.8%), Klebsiella pneumoniae (28.2%), Staphylococcus aureus (22.2%), and Pseudomonas aeruginosa (17.9%). VAP (45 patients), meningitis (9 patients) and sepsis/septic shock (47 patients) were diagnosed in 59 patients. Mortality was also higher in the group of patients who developed infectious complications (*p* < 0.001). The mean length of stay in the ICU was 17.8 ± 22.3 days and was longer in the mortality cohort (*p* = 0.002). The mean length of stay in the intensive care unit at the time of death was 25.3 ± 27 days. Brain death was detected in 18 (33.3%) patients. The most common cause of mortality was infective complications (41.7%). The other causes of mortality were intracranial pathologies related to the primary disease (39.5%), and serious cardiopulmonary events (18.8%).

**Figure 4:**
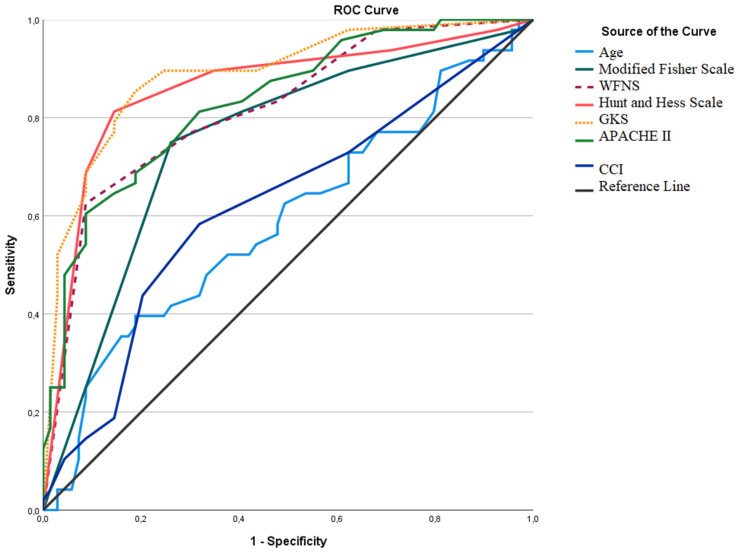
ROC analysis curves of quantitative parameters for mortality. WFNS: World Federation of Neurological Societies Scale, GCS: Glasgow Coma Scale, APACHE II: Acute Physiological and Chronic Health Evaluation II Score, CCI: Charlson Comorbidity Index.

Univariate logistic regression analysis was performed for the effect profiles of the parameters on mortality (Table 5). Multivariate logistic regression analysis was presented in Table 6. Loss of consciousness on admission, high CCI score, GCS score <8, H&H stage >2, meningitis and sepsis were found to be independent risk factors associated with mortality in multivariate and univariate regression analysis (Table 6). The presence of meningitis was closely associated with mortality and the mortality risk was 27.45 times higher in patients with meningitis compared with patients without meningitis. ROC analysis was performed to determine the predictive values and affect levels of parameters regarding mortality and the results are presented in Table 7 and Figure 4.

## 4. Discussion

Although patient outcomes have improved in recent years with advancing technology and treatment strategies, spontaneous SAH is still associated with high mortality and morbidity. Studies have reported that mortality in this patient group varies in a wide range of 8.3–66.7% and the mortality risk remains high after hospital discharge [20,21,22]. In our study, ICU mortality was 41% in patients with spontaneous SAH, which is similar to the rates reported in some recent studies [23,24] but is lower than the rates reported a decade ago [25,26]. Decreased mortality was most likely due to advances in follow-up/treatment in recent years. However, our results reveal higher mortality compared with some other reports [27,28]. The scores obtained from grading systems showing the clinical and radiological severity of the disease are actually the main factors predicting mortality in patients with spontaneous SAH [29,30,31,32]. Bohnstedt et al. stated that the H&H stage at presentation was the primary prognostic factor in determining patient outcome [33]. In the study by Waweru et al., 8.3% of the patient population had GCS <8 and 18.9% were at Modified Fisher Scale stage 4. In our study, 48.4% of our patient population were at Modified Fisher Scale stage 4, 30.8% at WFNS stage 5, 33.3% at H&H stage 5, and 40.2% had GCS <8, supporting the idea that the disease severity was higher compared with the patient population of the study reported by Waweru et al. [27]. The disease severity of the patient population in the study by Papadimitriou-Olivgeris et al., which evaluated mortality risk factors in spontaneous SAH patients followed in the ICU, was quite similar to that of our study, and the reported mortality (42%) was the same as that of our study [23]. Furthermore, in a recent study evaluating mortality in 137 patients with WFNS stage 5 who had spontaneous SAH, ICU mortality was 55%, hospital mortality was 64%, and brain death occurred in 60% of patients. WFNS stage 5 patients comprised the majority of patients with mortality, and brain death occurred in 60% of patients with WFNS stage 5 in our study [34]. Given this information, it is likely that the high mortality in our study sample was due to the high severity of spontaneous SAH. A recent study comparing the prognostic values of grading systems used in patients with aneurysmal SAH reported that WFNS stage ≥3.5 (75.9% sensitivity and 83% specificity) and H&H stage ≥3.5 (72.2% sensitivity and 84.4% specificity) were highly predictive of poor prognosis [35]. In another study investigating the effect of radiological scoring on prognosis in patients with SAH, it was reported that patients classified as being at Modified Fisher Scale stage 4 and H&H stage 5 were at high risk for mortality [36]. Similarly, Lantigua et al. have reported that APACHE II, GCS and Modified Fisher Scale scores on admission were important risk factors for mortality in patients with SAH [37]. In this study, WFNS stage ≥4. 5 (62.5% sensitivity and 91.3% specificity), H&H stage ≥3.5 (81.3% sensitivity and 85.5% specificity), GCS score ≤10.5 (85.4% sensitivity and 81.2% specificity), Modified Fisher Scale stage ≥3.5 (75% sensitivity and 73.9% specificity), and APACHE II score ≥18.5 (60.4% sensitivity and 91.3% specificity) were associated with an increased risk of mortality, and H&H stage >2 and GCS score <8 were found to be independent risk factors for mortality.

It is well known that ruptured intracranial aneurysms are the most common cause of spontaneous SAH and have worse prognoses than other causes of spontaneous SAH. However, incidence of aneurysms in spontaneous SAH patients varies between centres. Ramnarayan et al. reported that aneurysm was involved in the aetiology of 35.4% SAH patients in South India and that the incidence of aneurysm may show geographical differences [11]. A study from the USA analysing the aetiology of SAH in 6368 patients reported that 54% of patients had at least one aneurysm and 6% had arteriovenous malformations [38]. In studies from Turkey analysing the outcome of patients with spontaneous SAH, Özdemir et al. [39] reported that aneurysms were responsible for the aetiology of SAH in 72.7% of patients, while Küçük Pehlivanlar et al. [24] reported that aneurysms were responsible for the aetiology of spontaneous SAH in 91.3% of patients. A study analysing the incidence of spontaneous SAH in Turkey reported that aneurysms were found in 78.5% of patients with spontaneous SAH. In our study, angiography was positive in 72 (61.5%) of the patients (55.5% aneurysmatic and 6% arteriovenous malformation) and the rate of positive angiography was higher in the group of patients with mortality. Although the rate of aneurysmatic SAH was generally lower in our study compared with others reported from Turkey [24,39], it was still the most common aetiology. There are modifiable and non-modifiable risk factors in the aetiology of spontaneous SAH. Modifiable risk factors include smoking and alcohol consumption, hypertension and diabetes mellitus, whereas non-modifiable risk factors include gender, genetic factors and systemic rheumatological diseases [40]. Hypertension has been reported to be an important risk factor for the development and rupture of cerebral aneurysms, increasing the risk of spontaneous SAH sevenfold [41]. Despite the known association between hypertension and the development of spontaneous SAH, the effect of blood pressure changes in the acute phase of SAH on prognosis has not been clarified. Rosentgart et al. [42] have reported that high systolic blood pressure at presentation was an independent prognostic factor, whereas Gomis et al. [43] reported no association with prognosis. Duran et al. concluded that low blood pressure at presentation was associated with poor prognosis and mortality in patients with spontaneous SAH [44]. For the present study, the majority of patients were hypertensive at the time of ICU admission, and systolic blood pressure at ICU admission was not a significant determinant of mortality. Women are thought to be at greater risk of spontaneous SAH than men [40]. A systematic review and meta-analysis of 94,912 patients from 21 countries reported that this gender difference is associated with the absence of oestrogen and postmenopausal collagen depletion in women, particularly after the age of 50 [45]. Similarly, Rooij et al. have reported that the gender difference begins at the age of 55 [46]. Unlike a recent study investigating the effects of gender and age on spontaneous SAH, a generally higher incidence of SAH was found in males compared with females [47]. Furthermore, the effect of age and gender on patient outcomes was controversial. Some studies [48,49] report that increasing age and male gender are associated with poor prognosis, while others [33,43] suggest that they have no prognostic value. In our study, the majority of patients was male and no significant effect of age and gender on mortality was found. It is understandable that the predominance of female patients seen in some studies [50,51] was not seen in our study, given the mean age of male and female (premenopausal women) patients in our study. It is known that the most common presenting symptoms of spontaneous SAH patients are headache and loss of consciousness. Moreover, it has been shown in previous studies that loss of consciousness at the onset is an important indicator of severe haemorrhage and has prognostic value. In line with the literature, the most common presenting symptoms were headache (76.1%) and loss of consciousness (32.5%) in our patient cohort. Presentation with loss of consciousness was more frequent than headache in the mortality cohort. In addition, loss of consciousness was an independent risk factor for mortality in this study, with a 5.97-fold increase in mortality, according to univariate regression analysis.

In our study, the most common complications associated with spontaneous SAH were vasospasm (38.5%), hyponatraemia (35.9%), hydrocephalus (32.5%), DCI (30.8%), and the most common ICU-related complications were sepsis/septic shock (40.2%) and VAP (38.5%). Hydrocephalus, DCI, hypernatremia, meningitis, VAP and sepsis/septic shock complications were significantly higher in the mortality group, and meningitis and sepsis/septic shock were found to be independent risk factors for mortality. As spontaneous SAH is a complex disease that may involve multiple neurological injuries and systemic organ dysfunction, medical complications are common in patients with spontaneous SAH, especially in those with severe disease, and have a significant impact on outcome [1]. Infectious complications such as meningitis and VAP are known to contribute to poor outcomes in spontaneous SAH patients [5]. A study evaluating the prevalence of nosocomial infectious complications in patients with spontaneous SAH reported that pneumonia was the most common infection, that meningitis/ventriculitis was seen in 5% of patients, and that these infections both prolonged hospital stay and increased mortality in this patient group [52]. In parallel with this study, we found that 7.7% of patients developed meningitis and that mortality increased 27.45-fold in this patient group. Studies investigating risk factors for meningitis after intracranial surgery have reported that EVD, LD and major craniectomy are independent risk factors for the development of meningitis [53]. In our patient population, 27% required decompressive craniectomy, 53% required EVD, and 40.2% required lumbar drainage, with these interventions probably increasing the risk of meningitis. It has been recognised for many years that infectious complications increase with prolonged ICU stay and mechanical ventilation. In a study evaluating sepsis in patients with severe SAH in the ICU, the mean ICU stay was 17 (±17.3) days and sepsis was detected in 79.3% of patients [54]. Considering the duration of mechanical ventilation (14.1 ± 22 days) and ICU stay (17.8 ± 22.3 days) of our patients, the rate of sepsis/septic shock (40.2%) assumed to be within the expected range.

This study has several limitations. First, our study was a retrospective study based on data collected from hospital database systems. Secondly, only ICU outcomes were evaluated, and long-term neurological status and mortality were not included. Thirdly, due to the retrospective nature of the study, all factors that might affect mortality could not be evaluated. Finally, the fact that this study was performed in a single centre mean that our results may not be capable of reflecting the frequency of aneurysmal SAH.

## 5. Conclusions

Spontaneous SAH is a prevalent condition for those between the ages of 50 and 60 years, with a high mortality rate in severe cases. The findings of this study indicate that a history of loss of consciousness and the presence of septic complications are independent risk factors for the prediction of mortality. The findings of the present study demonstrate that the prevention of septic complications, assumed as an avoidable risk factor, is of significance in the reduction of mortality. Consequently, greater emphasis should be placed on healthcare-related efficacy metrics in this specific patient population, which may necessitate prolonged ICU hospitalization.

## Figures and Tables

**Table 1:** Clinical and demographic characteristics.

		Overall	Survival (*n* = 69, 59%)	Mortality (*n* = 48, 41%)	*p*
	Age, years	54.9 ± 15.1	52.8 ± 1.4	57.9 ± 15.8	0.072
Age group	Age <50 years	49 (41.9%)	32 (46.4%)	17 (35.4%)	0.237
	Age ≥50 years	68 (58.1%)	37 (53.6%)	31 (64.6%)	
Gender	Male	65 (55.6%)	34 (49.3%)	31 (64.6%)	0.101
	Female	52 (44.4%)	35 (50.7%)	17 (35.4%)	
Habits	Smoking	28 (23.9%)	20 (29%)	8 (16.7%)	0.125
	Alcohol abuse	6 (5.1%)	2 (2.9%)	4 (8.3%)	0.190
	Substance use	4 (3.4%)	3 (4.3%)	1 (2.1%)	0.507
Comorbidities	CCI	1 (0–9)	1 (0–7)	2 (0–9)	0.023
	Hypertension	75 (64.1%)	41 (59.4%)	34 (70.8%)	0.206
	Diabetes mellitus	22 (18.8%)	13 (18.8%)	9 (18.8%)	0.990
	Systemic rheumatological disease	2 (1.7%)	1 (1.4%)	1 (2.1%)	0.795
	Passed SAH	9 (7.7%)	5 (7.2%)	4 (8.3%)	0.828
	Autosomal dominant PKD	6 (5.1%)	4 (5.8%)	2 (4.2%)	0.694
Clinical and vital signs	Headache	89 (76.1%)	61 (88.4%)	28 (58.2%)	<0.001
	Neck stiffness	10 (8.5%)	4 (5.8%)	6 (12.5%)	0.202
	Neck pain	4 (3.4%)	1 (1.4%)	3 (6.3%)	0.160
	Loss of consciousness	38 (32.5%)	10 (14.5%)	28 (58.3%)	<0.001
	Nausea and vomiting	28 (23.9%)	17 (24.6%)	11 (22.9%)	0.830
	Seizure	14 (12%)	8 (11.6%)	6 (12.5%)	0.882
	Focal neurological deficit	7 (6%)	4 (5.8%)	3 (6.3%)	0.919
	SBP on admissionmmHg	140 (130–165)	140 (130–160)	140 (130–170)	0.824
	ICU admission time, hours	7 (4–11)	8 (4–14)	7 (3.5–9.5)	0.108

Abbreviations: CCI: Charlson Comorbidity Index, SAH: subarachnoid haemorrhage, PKD: polycystic kidney disease, SBP: systolic blood pressure, ICU: intensive care unit. Data are presented as mean ± standard deviation, minimum maximum and interquartile range (IQR) or n (%).

**Table 2:** Radiological data.

		Overall (*n* = 117)	Survival (*n* = 69, %59)	Mortality (*n* = 48, %41)	*p*
Angiography	Positive	72 (61.5%)	48 (69.5%)	24 (50%)	0.032
	Negative	45 (38.4%)	21 (30.4%)	24 (50%)	
Aetiology	Aneurysm	65 (55.6%)	42 (60.8%)	23 (47.9%)	0.110
	AVM	7 (6%)	6 (8.7%)	1 (2.1%)	
	Other	45 (38.5%)	21 (30.4%)	24 (50%)	
Modified Fisher Scale	Stage 1	28 (23.9%)	24 (34.8%)	4 (8.3%) ^b^	<0.001
	Stage 2	19 (16.2%)	15 (21.7%)	4 (8.3%) ^b^	
	Stage 3	13 (11.1%)	10 (14.5%) ^a^	3 (6.3%) ^b^	
	Stage 4	57 (48.4%)	20 (29%) ^a^	37 (77.1%) ^b^	
Aneurysm side	Right	32 (27.4%)	21 (30.4%)	11 (22.9%)	0.280
	Left	24 (20.5%)	17 (24.6%)	7 (14.6%)	
	Both sides	9 (7.7%)	4 (5.8%)	5 (10.4%)	
Aneurysm location	Saptanmadı	52 (44.4%)	27 (39.1%)	25 (52.1%)	0.353
	ICA	10 (8.5%)	7 (10.1%)	3 (6.3%)	
	ACA	29 (24.8%)	19 (27.5%)	10 (20.8%)	
	MCA	16 (13.7%)	12 (17.4%)	4 (8.3%)	
	BA	6 (5.1%)	2 (2.9%)	4 (8.3%)	
	PCoA	4 (3.4%)	2 (2.9%)	2 (4.2%)	
Aneurysm size	Not detected	52 (44.4%)	27 (39.1%)	25 (52.1%)	0.434
	<5 mm	25 (21.4%)	18 (26.1%)	7 (14.6%)	
	5–9,9 mm	26 (22.2%)	17 (24.6%)	9 (18.8%)	
	10–24,9 mm	11 (9.4%)	6 (8.7%)	5 (10.4%)	
	≥25 mm	3 (2.6%)	1 (1.4%)	2 (4.2%)	

Abbreviations: AVM: arteriovenous malformation, ICA: internal carotid artery, ACA: anterior cerebral artery MCA: middle cerebral artery, BA: basilar artery, PCoA: posterior communicating artery. In the multiple mixed matrix, proportions that are statistically significantly different between proportional values between columns are labelled (a) and (b).

**Table 3:** Scoring system data.

	Scores	Overall (*n* = 117)	Survival (*n* = 69, %59)	Mortality (*n* = 48, %41)	*p*
	GCS	11 (6–14)	14 (12–15)	5 (8–3)	<0.001
GCS (group)	3–8	47 (40.2%)	10 (14.5%)	37 (77.1%)	<0.001
	9–12	18 (15.4%)	12 (17.4%)	6 (12.5%)	
	13–15	52 (44.4%)	47 (68.1%)	5 (10.4%)	
	APACHE II	14 (2–35)	11 (2–29)	22 (6–35)	<0.001
	WFNS	3 (2–5)	2 (1–4)	5 (4–5)	<0.001
WFNS (distribution)	Stage 1	23 (19.7%)	22 (31.9%) ^a^	1 (2.1%) ^b^	<0.001
	Stage 2	21 (17.9%)	14 (20.3%)	7 (14.6%)	
	Stage 3	15 (12.8%)	12 (17.4%)	3 (6.3%)	
	Stage 4	22 (18.8%)	15 (21.7%)	7 (14.6%)	
	Stage 5	36 (30.8%)	6 (8.7%) ^a^	30 (62.5%) ^b^	
	H&S	3 (2–5)	2 (1–3)	5 (4–5)	<0.001
H&S (distribution)	Stage 1	17 (14.5%)	15 (21.7%) ^a^	2 (4.2%) ^b^	<0.001
	Stage 2	27 (23.1%)	25 (36.2%) ^a^	2 (4.2%) ^b^	
	Stage 3	18 (15.4%)	14 (20.3%)	4 (8.3%)	
	Stage 4	10 (8.5%)	4 (5.8%)	6 (12.5%)	
	Stage 5	45 (38.4%)	11 (15.9%) ^a^	34 (70.9%) ^b^	

Abbreviations: GCS: Glasgow Coma Scale, APACHE II: Acute Physiological and Chronic Health Evaluation II score, WFNS: World Federation of Neurological Societies, H&S: Hunt and Hess. In the multiple mixed matrix, proportions that are statistically significantly different between proportional values between columns are labelled (^a^) and (^b^). Data are presented as mean ± standard deviation, minimum maximum and interquartile range (IQR) or n (%).

**Table 4:** Treatments and complications.

		Overall (*n* = 117)	Survival (*n* = 69, %59)	Mortality (*n* = 48, %41)	*p*
*Treatment*	Endovascular coil	6 (5.1%)	4 (5.8%)	2 (4.2%)	0.353
	Neurosurgery	79 (67.5%)	43 (62.3%)	36 (75%)	
	Conservative	32 (27.4%)	22 (31.9%)	10 (20.8%)	
*Complications*	Vasospasm	45 (38.5%)	22 (31.9%)	23 (47.9%)	0.080
	Rebleeding	19 (16.2%)	8 (11.6%)	11 (22.9%)	0.102
	Hydrocephalus	38 (32.5%)	14 (20.3%)	24 (50%)	<0.001
	DCI	36 (30.8%)	15 (21.7%)	21 (43.8%)	0.011
	Hyponatraemia	42 (35.9%)	22 (31.9%)	20 (41.7%)	0.278
	Hypernatraemia	30 (25.6%)	11 (15.9%)	19 (39.6%)	0.004
	Seizure	12 (10.3%)	8 (11.6%)	4 (8.3%)	0.567
	Pulmonary oedema	31 (26.5%)	13 (18.8%)	18 (37.5%)	0.024
	Meningitis	9 (7.7%)	1 (1.4%)	8 (16.7%)	0.002
	VAP	45 (38.5%)	14 (20.3%)	31 (64.6%)	<0.001
	Sepsis/Septic shock	47 (40.2%)	15 (21.7%)	32 (66.7%)	<0.001
	Microbiological culture (+)	59 (50.4%)	26 (68.8%)	33 (37.7%)	<0.001
*Additional treatments*	Vasopressor (+)	50 (42.7%)	11 (15.9%)	39 (81.3%)	<0.001
	Controlled hypertension (+)	12 (10.3%)	5 (7.2%)	7 (14.6%)	0.318
*Drainage system*	EVD	62 (53%)	43 (62.3%) ^a^	19 (39.6%) ^b^	0.002
	LD	47 (40.2%)	19 (27.5%) ^a^	28 (58.3%) ^b^	
	Yok	8 (6.8%)	7 (10.1%)	1 (2.1%)	
	Decompressive craniectomy	32 (27.4%)	10 (14.5%)	22 (45.8%)	<0.001
	Duration of mechanical ventilation, day	14.1 ± 22	7.4 ± 15.7	23.6 ± 26	<0.001
	Length of stay in the ICU, day	17.8 ± 22.3	12.5 ± 16.6	25.3 ± 27	0.002

Abbreviations: DCI: delayed cerebral ischaemia VAP: ventilator-associated pneumonia, EVD: extraventricular drainage catheter LD: lumbar drainage catheter, ICU: intensive care unit. In the multiple mixed matrix, proportions that are statistically significantly different between proportional values between columns are labelled (^a^) and (^b^). Data are presented as mean ± standard deviation, minimum maximum and interquartile range (IQR) or n (%).

**Table 5:** Univariate logistic regression analysis for parameters.

Variables	Univariate		
	OR	95% CI	*p*
Age	1.02	0.998–1.049	0.074
Gender	1.31	0.41–4.17	0.640
CCI	1.52	1.04–2.23	0.029
GCS (<8)	0.10	0.02–0.54	0.007
Loss of consciousness	5.97	1.76–20.20	0.004
Modified Fisher Scale	0.49	0.09–2.59	0.404
H&S (>2)	5.32	1.07–26.43	0.040
WFNS	0.46	0.07–2.74	0.400
DCI	2.10	0.58–7.64	0.256
Hydrocephalus	1.73	0.50–6.00	0.382
Vasospasm	1.965	0.920–4.201	0.081
Meningitis	13.6	1.640–112.764	0.016
Sepsis/Septic shock	5.24	1.36–20.19	0.015
Rebleeding	2.39	0.52–10.9	0.258
Decompressive craniectomy	0.83	0.20–3.31	0.793

Abbreviations: CCI: Charlson Comorbidity Index, GCS: Glasgow Coma Scale, WFNS: World Federation of Neurological Societies, H&S: Hunt and Hess, DCI: delayed cerebral ischaemia, OR: odds ratio, CI: confidence interval.

**Table 6:** Multivariate logistic regression analysis for parameters.

Variables	Multivariate Model ^†^			*p*
	Nagelkerke R^2^ = 0.637			
	OR	95% CI		
		Lower Level	Upper Level	
CCI	1.294	1.173	1.427	<0.001
GCS (<8)				
3–8	7.289	3.590	14.802	<0.001
9–12	1.853	0.947	3.626	0.072
13–15 (ref)				
Loss of consciousness				
Yes	4.438	2.718	7.246	<0.001
No (ref)				
H&S (>2)				
Score >2	4.324	2.227	8.395	<0.001
Score ≤2 (ref)				
Meningitis				
Yes	27.452	7.297	103.279	<0.001
No (ref)				
Sepsis/Septic shock				
Yes	3.442	2.224	5.327	<0.001
No (ref)				

Abbreviations: CCI: Charlson Comorbidity Index, GCS: Glasgow Coma Scale, H&S: Hunt and Hess, OR: odds ratio, CI: confidence interval, ref: reference category. ^†^ Analysis has been carried out with a virtual dataset generated with a non-parametric bootstrapping method with a confidence interval (CI) of 95%. A virtual database (with a sample size of 1000) is generated with the Python programming language using IIDBootstrap (the libraries used are pandas and numpy).

**Table 7:** ROC analysis and cut-off values of quantitative parameters formortality.

	AUC (%95 CI)	Cut-Off	*p*	Sensitivity (%)	Specificity (%)
Age	0.589 (0.482–0.696)	63.5	0.104	39.6%	81.2%
Modified Fisher Scale	0.752 (0.660–0.843)	≥3.5	<0.001	75.0%	73.9%
WFNS	0.818 (0.739–0.896)	≥4.5	<0.001	62.5%	91.3%
H&S	0.859 (0.783–0.934)	≥3.5	<0.001	81.3%	85.5%
GCS	0.887 (0.824–0.950)	≤10.5	<0.001	85.4%	81.2%
APACHE II	0.831 (0.757–0.906)	≥18.5	<0.001	60.4%	91.3%
CCI	0.620 (0.515–0.726)	≥1.5	0.027	58.3%	68.1%

Abbreviations: CCI: Charlson Comorbidity Index, GCS: Glasgow Coma Scale, WFNS: World Federation of Neurological Societies, H&S: Hunt and Hess, APACHE II: Acute Physiological and Chronic Health Evaluation II score, AUC: area under the curve, CI: confidence interval.

## Data Availability

All data generated or analysed during this study are included in this article. Further inquiries can be directed to the corresponding author.
